# The reduction in FOXA2 activity during lung development in fetuses from diabetic rat mothers is reversed by Akt inhibition

**DOI:** 10.1002/2211-5463.12517

**Published:** 2018-09-25

**Authors:** Qingmiao Zhang, Xinqun Chai, Feitao Deng, Weixiang Ouyang, Ting Song

**Affiliations:** ^1^ Department of Obstetrics and Gynecology Union Hospital Tongji Medical College Huazhong University of Science and Technology Wuhan China; ^2^ Department of Hepatobiliary Surgery Union Hospital Tongji Medical College Huazhong University of Science and Technology Wuhan China

**Keywords:** Akt, fetal lung development, FOXA2, maternal diabetes mellitus, surfactant protein B, surfactant protein C

## Abstract

Hyperglycemia during pregnancy is associated with fetal lung development disorders and surfactant protein (SP) deficiency. Here, we examined the role of FOXA2 and Akt signaling in fetal lung development during diabetic pregnancy. Sprague‐Dawley rats were injected with streptozocin (STZ) during pregnancy to induce diabetes (DM). DM‐exposed fetal lungs exhibited reduced numbers of alveoli, irregularities in the appearance and thickness of the alveolar septum, increased levels of glycogen and lipids in type II alveolar epithelial cells, fewer microvilli and mature lamellar bodies, and swollen mitochondria. SP‐B and SP‐C in DM amniotic fluid and DM lungs were lower than in the control group (*P* < 0.05). DM lung nuclear FOXA2 was lower compared with the control group (*P* < 0.05), but p‐FOXA2 was higher (*P* < 0.05). In murine lung epithelial (MLE) 12 cells, p‐AKT levels were increased by high glucose/insulin, but decreased by the Akt inhibitor MK2206 (*P* < 0.05). Expression of nuclear FOXA2 was increased by MK2206 compared with the high glucose/insulin group (*P* < 0.05). These results suggest that maternal diabetes induces fetal lung FOXA2 phosphorylation through the Akt pathway, and also affects the maturation of alveolar epithelial cells and reduces levels of SP‐B and SP‐C in the fetal lungs. An Akt inhibitor reversed the changes in SP expression *in vitro*.

**Abbreviations**
AECII: type II alveolar cells
DM: diabetes mellitus
MLE 12: murine lung epithelial 12
RDS: neonatal respiratory distress syndrome
SP: surfactant protein
STZ: streptozocin

Hyperglycemia [either maternal diabetes or gestational diabetes mellitus (DM)] during pregnancy is associated with adverse maternal and fetal outcomes 1, 2. Poor control of blood glucose in mothers may lead to fetal lung development disorders and surfactant protein (SP) deficiency 3. Deficiency of alveolar SPs along with structural immaturity of the lung may lead to neonatal respiratory distress syndrome (RDS), which is associated with high mortality 4. The natural course of RDS begins at birth (or shortly after) and increases in severity over the first 2 days of life. Lung development requires the coordination and completion of five developmental stages (embryonic period, pseudoglandular period, canalicular period, original saccular period, and alveolar stages) 5, 6, 7. These processes involve both structural maturation and SP expression.

The SPs are components of the surfactant and host defense systems required for postnatal adaptation to air breathing 2, 8. They are part of the surface‐active lipoprotein complex formed by type II alveolar cells (AECII) 9 and reduce surface tension, facilitating gas exchange 9. SP‐B is essential for lung function and homeostasis after birth 10. SP‐C is extremely hydrophobic and plays an essential role in the formation of the SP complex for normal lung function 11.

Lung maturation is influenced by many transcription factors, growth factors, and signaling molecules 5, 6, 7. The Fox family of transcription factors has wide functions in embryonic development, cell cycle regulation, glucose and lipid metabolism, aging, and immune regulation 12, 13. Forkhead box A2 (FOXA2), also called hepatocyte nuclear factor 3B, regulates lung and pancreas development and glucose and lipid metabolism and is mainly expressed in the liver, pancreas, lung, and adipose tissue 8. FOXA2 is involved in diabetes and response to hyperglycemia by regulating alpha‐cell differentiation and glucagon secretion 12. In the lung, FOXA2 expression is restricted to specific subsets of respiratory epithelial cells 14 and is limited to AECII late in gestation, where it plays an important role in alveolar epithelial cell differentiation and SP expression 15. FOXA2 is coexpressed with a number of proteins involved epithelial cell differentiation and lung development, including thyroid transcription factor 1, Clara cell secretory protein, Muc5A/C, E‐cadherin, vascular endothelial growth factor A, SP‐A, SP‐B, SP‐C, and SP‐D 13, 14, 15, 16, 17, 18, 19, 20, 21, 22. FOXA2 plays essential roles at multiple important periods of lung morphogenesis 23 and is important for TH2 cell‐mediated innate immunity in the developing lungs 24. FOXA2 can regulate the differentiation of AECII and the synthesis of pulmonary surfactants 2, 16.

Energy homeostasis is controlled via the insulin receptor and insulin substrate and involves various downstream pathways such as phosphoinositide‐3‐kinase (PI3K)/Akt and mitogen‐activated protein kinase 3/1 (MAPK3/1). The major metabolic effector of insulin is thought to be the PI3K/Akt pathway, which contributes to metabolic control 25. PI3K/Akt signaling has a very wide range of downstream effects and is also thought to play a pivotal role in mouse lung development 26. Akt/PKB can phosphorylate FOXA2 directly, resulting in p‐FOXA2 being released from the nucleus into the cytoplasm 27, inhibiting the transcriptional activity of FOXA2 28. This inactive form of FOXA2 can be regulated by insulin receptors and Akt in the liver of mice with insulin resistance and hyperinsulinemia 29.

As a consequence of diabetic pregnancy, the fetus is exposed to hyperglycemia and shows responsive fetal hyperinsulinemia 30. We hypothesized that fetal diabetes exposure will promote the phosphorylation of FOXA2, leading to decreased FOXA2 transcription activity in AECII and fetal lung development disorders. Therefore, the aim of the present study was to explore the impact of diabetes exposure on lung development and the events affecting the function of FOXA2 in the lung of rats from mothers with diabetes and to analyze whether the related regulation is mediated by the Akt signaling pathway.

## Materials and methods

### Animals

Specific pathogen‐free grade adult Sprague‐Dawley rats (female = 20, male = 10, 220–240 g) were purchased from the Hubei Province Center for Disease Control and Prevention and housed at the Animal Experiment Center of Tongji Medical College. Virgin female rats were mated with normal males (female: male ratio of 2 : 1). On the day of conception (day 0 of pregnancy), the presence of sperm in vaginal smears was recorded as pregnancy. Ten pregnant female rats received a single intraperitoneal injection of streptozocin (STZ; 45 mg·kg^−1^; Sigma, St Louis, MO, USA) on day 2 (DM group). The rats were determined as having diabetes according to fasting blood glucose levels > 6.1 mmol·L^−1^ over three consecutive measurements 31. All 10 rats developed diabetes, and there was no need for replacement. The remaining ten control mothers received saline injection instead of STZ. The fetal lung maturation period was defined based on a previous report, which showed well‐differentiated AECII expressing SP‐B and SP‐C 2. The study was approved by the Institutional Animal Care and Use Committee, Tongji Medical College, Huazhong University of Science and Technology (No. S607).

### Sample collection

Nonfasting blood glucose was measured in the morning from blood collected from the tail vein and using a blood glucose meter. Nonfasting serum insulin levels were measured on day 21 after rats were anesthetized to collect the blood from the heart. Rat fetuses were taken from the uterus after the mothers were sacrificed by cervical dislocation under anesthesia (10% chloral hydrate, 3 mL·kg^−1^, intraperitoneally) on day 21. Fetal blood, amniotic fluid, and fetal lung samples were taken. Parts of the fetal lungs were fixed in 10% formalin, processed, embedded in paraffin, and sectioned. Sections were stained with hematoxylin and eosin for histological examination. Parts of the fetal lungs were fixed in 0.1 mmol·L^−1^ glutaraldehyde for electronic microscopy.

### Cell culture and treatment

MLE12 (mouse lung epithelial type II cells) were purchased from the American Type Culture Collection (ATCC, Manassas, VA, USA). Cells were cultured in Dulbecco's modified Eagle's medium (DMEM; low glucose, Hyclone, Thermo Fisher Scientific, Waltham, MA, USA) containing 10% fetal bovine serum (GIBCO, Invitrogen Inc., Carlsbad, CA, USA) in 25‐cm^2^ plastic flasks (Corning Inc., Corning, NY, USA). Cells were used at passages three to six. Plastic flasks were kept under 5% CO_2_ at 37 °C. When the cells were 70–80% confluent, they were trypsinized with 0.05% trypsin/EDTA (Hyclone, Thermo Fisher Scientific) and resuspended in complete media. Resuspended cells were seeded in 6‐well plates. In order to study the effects of insulin resistance on FOXA2, an insulin resistance cell model was induced by different conditions of insulin and glucose [no treatment; 30 mm glucose and 25 nm insulin; 30 mm glucose; 25 nm insulin; and 5 μm MK22062·2HCl (Selleckchem, Houston, TX, USA)]. These conditions have been shown to mimic the condition of insulin resistance in cells 32, 33. The cells were harvested after 48 h of incubation for the subsequent experiments.

### Transmission electronic microscopy

Tissue specimens (< 1 mm^3^) were fixed in 2.5% glutaraldehyde in phosphate buffer for at least 3 h. Specimens were rinsed thrice with 0.1 m phosphate buffer. Blocks were fixed with 1% osmium acid for 2–3 h and rinsed thrice with 0.1 m phosphate buffer. Blocks were dehydrated in 50% ethanol for 15–20 min, 70% ethanol for 15–20 min, 90% ethanol for 15–20 min, 90% ethanol and 90% acetone (1 : 1) for 15–20 min, and 90% acetone for 15–20 min, all at 4 °C. Blocks were incubated in 100% acetone at room temperature for 15–20 min. For embedding, blocks were incubated in pure acetone and embedding solution (2 : 1) at room temperature for 3–4 h, in pure acetone and embedding solution (1 : 2) at room temperature overnight, and in pure embedding solution at 37 °C for 2–3 h. Blocks were dried at 37 °C overnight, 45 °C for 12 h, and 60 °C for 24 h. A LKB‐1 ultra‐thin slicer was used to obtain 50‐ to 60‐nm‐thick sections. Sections were treated with 3% uranyl‐lead citrate double staining and observed under a JEM‐1200EX microscope (JEOL, Tokyo, Japan).

### Paraffin‐embedded sections

Fresh tissue specimens were fixed in 4% paraformaldehyde for > 24 h (the same volume was used for each specimen). Blocks were treated with alcohol gradient for dehydration and embedded in paraffin. Blocks were cut to obtain 4‐μm sections. Sections were treated at 60 °C and stained with hematoxylin and eosin (HE) to observe the structure of the lungs.

### Immunohistochemistry

Samples of fetal lungs were taken from three different litters from each group by random sampling (*n* = 3/group). Paraffin sections were placed in an oven at 65 °C for 2 h, dewaxed, and washed three times with PBS (5 min each time). Antigens were retrieved in EDTA buffer in a low‐power microwave until boiling. After natural cooling, sections were washed thrice with PBS (5 min each time). Peroxidase was eliminated with 3% hydrogen peroxide solution at room temperature for 10 min. Sections were washed in PBS thrice, 5 min each time, and treated with 5% BSA for 20 min. Sections were stained with 100 μL (1 : 200) of diluted primary antibody against SP‐B (ABS21; Millipore corp., Billerica, MA, USA) or SP‐C (sc‐13979; Santa Cruz Biotechnology, Santa Cruz, CA, USA) at 4 °C overnight. After washing with PBS thrice (5 min each time), 100 μL (1 : 200) of secondary antibody (HRP‐labeled goat anti‐rabbit, AS‐1107) was added and incubated at 37 °C for 50 min. Antibodies were revealed with 100 μL of fresh DAB solution. The sections were observed by light microscopy (Olympus, Tokyo, Japan).

### Immunofluorescence

Samples of fetal lungs were taken from each group by random sampling. Paraffin sections were prepared as for immunohistochemistry. The diluted primary antibody (100 μL; 1 : 100; anti‐HNF3β/FOXA2; Millipore corp.), TdT, and dUTP (1 : 9, for TUNEL assay) were added and incubated at 4 °C overnight. After washing in PBS thrice (5 min each time), 100 μL (1 : 50) of each secondary antibody was added (FITC goat anti‐rabbit; AS‐1110, for FOXA2) and incubated at 37 °C for 50 min. The sections were counterstained with 4′,6‐diamidino‐2‐phenylindole and observed under fluorescence microscopy (Olympus).

### Real‐time reverse‐transcription polymerase chain reaction

Total mRNA was extracted from frozen fetal lung tissue and cells of each group (*n* = 3) using TRIZOL (Invitrogen Inc.) and reverse‐transcribed using the RT reagent kit (Takara Bio, Otsu, Japan). Real‐time PCR was performed using the Bio‐Rad CFX96 Detection System (Bio‐Rad, Hercules, CA, USA) with SYBR‐green (Bio‐Rad). PCR conditions were (a) 95 °C for 3 min and (b) 35 cycles at 95 °C for 10 s, 60 °C for 45 s, 95 °C for 39 s, and 60 °C for 15 s. Each sample was tested in triplicate, and the mean values were used for quantification. Relative gene expression profiles were analyzed by normalizing to β‐actin using the 2^−(ΔΔCt)^ method. The primers are listed in Table 1.

**Table 1:** Primers for Real‐time PCR

Gene	Forward (5′–3′)	Reverse (5′–3′)
R‐FOXA2	TGAAGCCCGAGCACCATTAC	CCA GGG TAG TGC ATG ACC TGT T
R‐SP‐B	AAAGCCTGGAGCAAGCGATAC	GAAAGCGTCTTCCTTGGTCATC
R‐SP‐C	TTGTCGTCGTGGTGATTGTAGG	GAAGGTAGCGATGGTGTCTGTG
R‐Actin	CGTTGACATCCGTAAAGACCTC	TAGGAGCCAGGGCAGTAATCT
M‐GAPDH	TGA AGG GTG GAG CCA AAAG	AGTCTTCTGGGTGGCAGTGAT
M‐SP‐B	GGCTAAGCCAGA ACA GAATCC	AGA AGTCCTGAGTGTGAGGCC
M‐SP‐C	CGTTGTCGTGGTGATTGTAGG	AAAGGTAGCGATGGTGTCTGC

R, rat; M, mouse.

### Western blot

Total proteins, cytoplasmic proteins, and nuclear proteins were extracted from frozen fetal lung tissue and cells using the Total Protein Extraction Kit and the Nuclear and Cytoplasmic Protein Extraction Kit (Beyotime Institute of Biotechnology, Haimen, China), according to the manufacturer's instructions. After heat denaturation at 100 °C for 3–5 min, 45 μg of protein was subjected to polyacrylamide gel electrophoresis (SDS/PAGE) and transferred to polyvinylidene fluoride (PVDF) membranes (Bio‐Rad). The membranes were blocked in 5% skim milk/TBST (Tris‐buffered saline with Tween 20) for 1 h and incubated overnight at 4 °C with antibodies (anti‐FOXA2 antibody; sc‐6554; Santa Cruz, CA, USA; 1 : 1000; anti‐Akt antibody; #4691; Cell Signaling, Danvers, MA, USA; 1 : 1500; anti‐p‐Akt antibody; #4060, Cell Signaling; 1 : 1000; anti‐GAPDH; #AF6276; Santa Cruz Biotechnology; 1 : 10 000; anticaspase 3; #9664, Cell Signaling; and anti‐PARP‐1; #9532, Cell Signaling). After washing twice in TBST, the membranes were incubated with peroxidase‐conjugated goat anti‐rat IgG (1 : 10 000) and washed in TBST twice. Immunoreactivity was detected using an enhanced electrochemiluminescence (ECL) kit and visualized in ImageJ.

### Immunoprecipitation

Nuclear proteins from fetal lungs of each group or MLE12 cells from each treatment group were purified using the NE‐PER Nuclear and Cytoplasmic Extraction Reagents (#78835; Thermo Fisher Scientific). FOXA2 was immunoprecipitated using an anti‐FOXA2 antibody (sc‐6554; Santa Cruz Biotechnology) immobilized on protein A/G Agarose (Beyotime Institute of Biotechnology). Phosphorylated FOXA2 (p‐FOXA2) was analyzed by western blot using antibody against phospho‐threonine (SC‐5267; Santa Cruz Biotechnology).

### Enzyme‐linked immunosorbent assay

Commercial ELISA kits (Elabscience Biotechnology, China; coefficient of variation < 10%) were used to detect secreted SP‐B and SP‐C in the amniotic fluid and insulin in the serum. All assays were performed with four separate samples in duplicate. Plates were read at 450 nm on a microplate ELISA reader.

### Statistical analysis

Statistical analysis was performed using graphpad prism 5 (GraphPad Software Inc., San Diego, CA, USA). Data from representative experiments were presented as means ± standard deviations. Comparisons of two groups were performed using the Student *t*‐test. Multiple groups were compared with ANOVA. *P* < 0.05 was considered to be significant.

## Results

### Rat models of maternal diabetes

Female rats were randomized to *n* = 10 dams/litters per group. Two diabetic (one with miscarriage on day 6 and the other with growth arrest on day 8) and one control (one case of intrauterine fetal death was found in the control group, which might have been due to anesthesia) litters were excluded from analysis due to pregnancy loss, leaving *n* = 8 dams/73 pups in the DM group and 9 dams/113 pups in the control group. The mothers were monitored for urine output during pregnancy; no differences were observed between the two groups. Litter size was lower in the DM group (9 ± 1 vs. 14 ± 1, *P* < 0.05 vs. the control group) (Table 2). All offspring of both genders were included in the analysis, but sex was not recorded.

**Table 2:** Maternal and fetal characteristics of rats with and without diabetes exposed (DM). The DM group had fewer offspring and lower average birth weight than the control group

Groups	Fetal viability (%)	Fetal number	Fetal weight (g)	Maternal glucose (mmol·L^−1^)	Fetal glucose (mmol·L^−1^)	Maternal insulin (pg·mL^−1^)	Fetal insulin (pg·mL^−1^)
DM (*n* = 8)	82	9.13 ± 1.04*	3.07 ± 0.03*	29.64 ± 0.93*	17.38 ± 0.65*	1864.0 ± 149.1*	729.1 ± 58.0*
Control (*n* = 9)	88	14.13 ± 0.88	3.54 ± 0.06	8.275 ± 0.62	2.90 ± 0.16	675.6 ± 119.5	564.1 ± 35.7

Maternal and fetal rats in the DM group showed significantly higher levels of blood glucose and blood insulin compared with rats in the control group (*P <* *0.05). Glucose and insulin were measured at 21 days.

### DM mothers and offspring had higher blood glucose and insulin levels

Diabetes mellitus pregnant rats showed significantly higher nonfasting blood glucose levels on day 21 compared with controls (29.6 ± 0.9 vs. 8.3 ± 0.6 mmol·L^−1^, *P *<* *0.05). In addition, the DM rat fetuses had higher blood glucose levels (17.4 ± 0.6 vs. 2.9 ± 0.2 mmol·L^−1^, *P *<* *0.05 vs. controls). Comparison of blood insulin levels on day 21 showed significantly higher insulin levels in the DM group (1864 ± 149 vs. 676 ± 120 pg·mL^−1^, *P *<* *0.05 vs. controls). The DM offspring also suffered from higher blood insulin levels (729 ± 58 vs. 564 ± 36 pg·mL^−1^, *P *<* *0.05 vs. controls) (Table 2). It should be noted that fetal blood was sampled after maternal chloral hydrate anesthesia and sacrifice.

### DM rat fetuses showed lung pathologic and cellular evidence of immaturity

**Figure 1:**
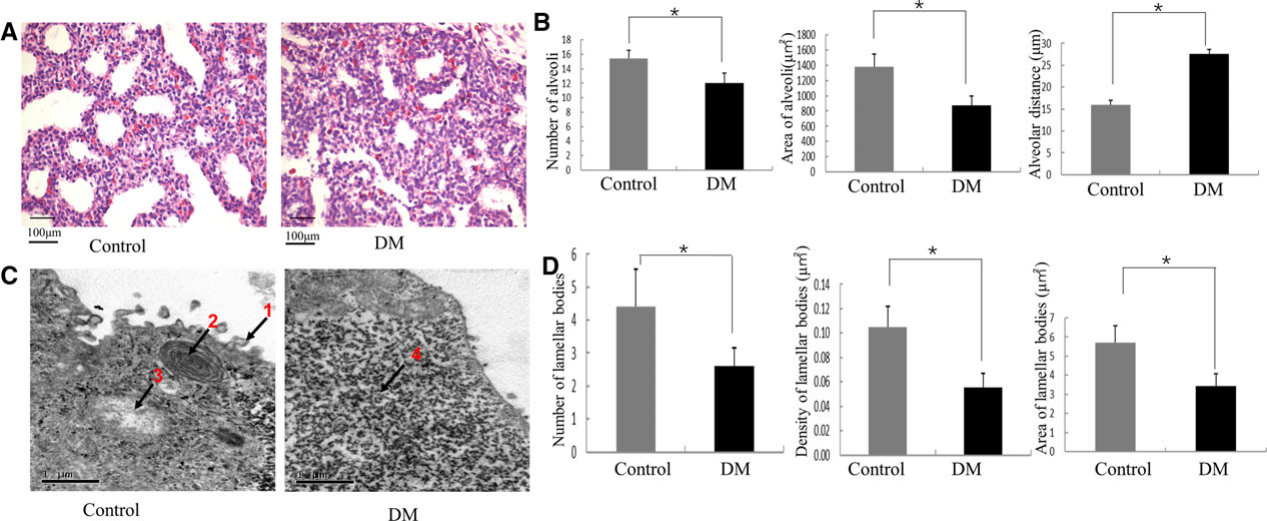
Gross morphology and ultrastructure of fetal rat lung. (A) Fetal rat lungs from the diabetes and control groups were stained with H&E (200× magnification), *n* = 5/group. (B) Five sections were examined and quantified to analyze the number, area, and distance of alveoli, as well as the number, area, and density of lamellar bodies. (C) The structure of fetal lung tissue was observed by electron microscopy, *n* = 5/group. (D) Lamellar bodies were analyzed by transmission electron microscopy. Bars represent means ± SD; *P < 0.05. Arrow 1: microvilli; Arrow 2: mature lamellar bodies; Arrow 3: mitochondria; Arrow 4: glycogen.

Diabetes mellitus fetal lung tissues showed a reduced number of alveoli, irregular appearance, and irregular thickness of the alveolar septum under light microscopy (Fig. 1A,B). Under electron microscopy, more glycogen and lipid in AECII were observed in DM fetal lungs, with few microvilli, few mature lamellar bodies, and swollen mitochondria (Fig. 1C,D).

### SP‐B and SP‐C levels were lower in the DM amniotic fluid and DM fetal lungs

**Figure 2:**
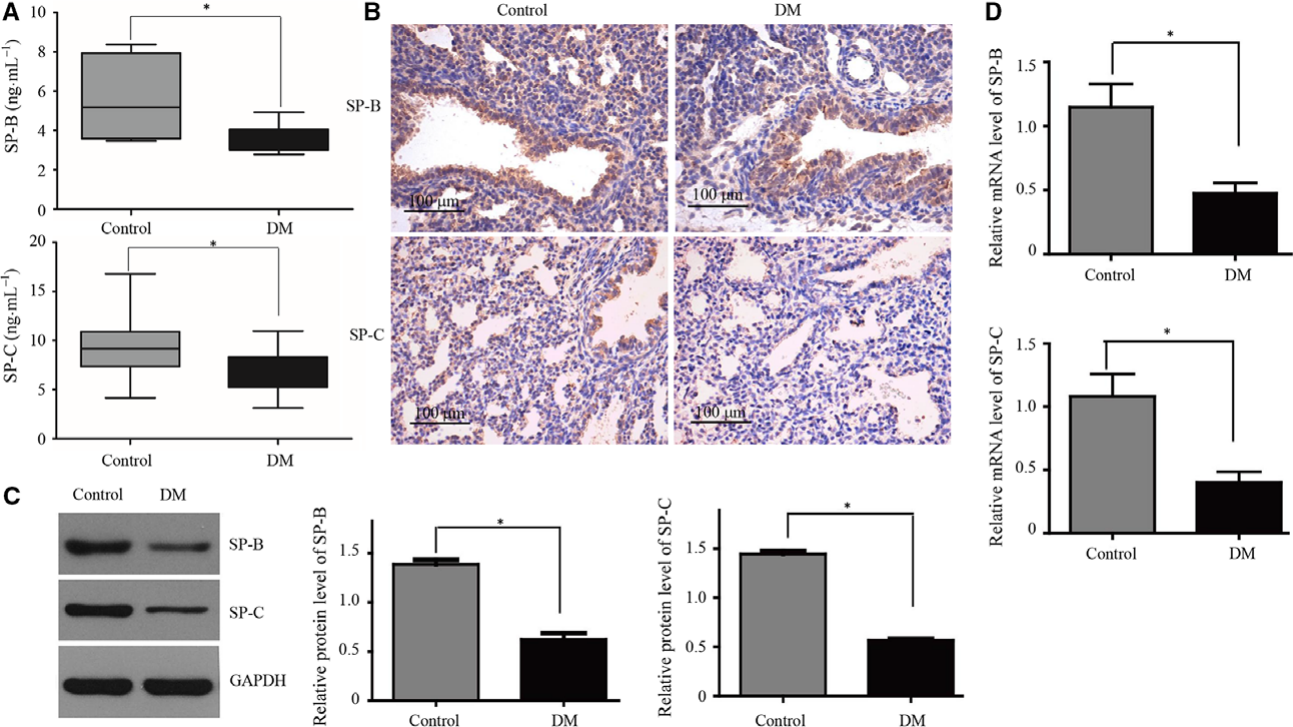
Expression levels of SP‐B and SP‐C in amniotic fluid and fetal lungs. On day 21, maternal rats in the diabetes and control groups were sacrificed. Amniotic fluid and fetal lungs were taken. (A) Levels of SP‐B and SP‐C in amniotic fluid by ELISA,* n* = 5/group. (B) Expression of SP‐B and SP‐C in fetal lung tissues by immunohistochemistry, *n* = 5/group. Scale bar, 100 μm. (C) Levels of SP‐B and SP‐C in fetal lungs by western blot. (D) mRNA levels of SP‐B and SP‐C in fetal lungs by RT‐PCR. Bars represent means ± SD; *P <* *0.05 between indicated samples.

The expression of SP‐B and SP‐C in the DM amniotic fluid was lower than in controls (*P *<* *0.05) (Fig. 2A). SP‐B and SP‐C were observed in AECII in the control and DM groups by immunohistochemistry (Fig. 2B). In fetal lung tissues, the protein and mRNA expressions of SP‐B and SP‐C in the DM fetal lungs were lower than in controls (*P *<* *0.05) (Fig. 2C,D). These results indicate that SP‐B and SP‐C protein and mRNA expressions were lower in the DM rat fetal lungs.

### DM fetal lungs showed a lower expression of nuclear FOXA2 and a higher expression of p‐FOXA2

**Figure 3:**
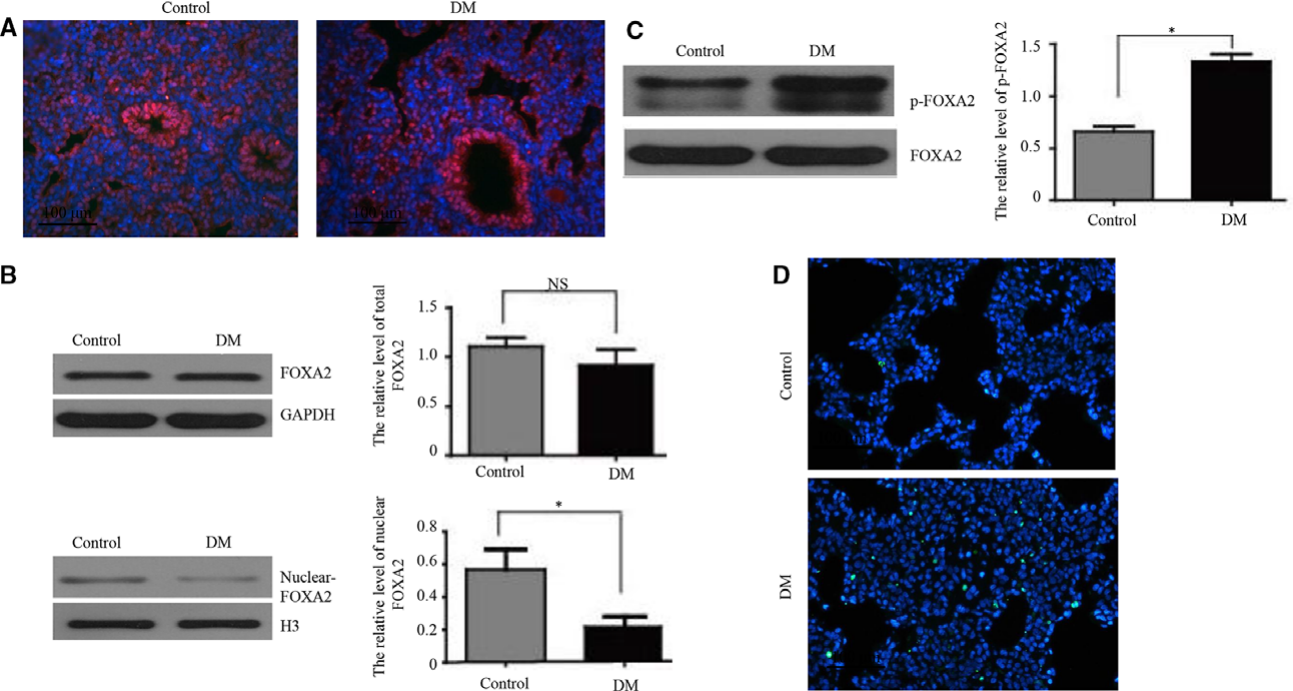
Expression of FOXA2, nuclear FOXA2, and p‐FOXA2 in fetal lungs. (A) Detection of FOXA2 in fetal lungs by immunofluorescence. Scale bar, 100 μm. *n* = 3/group. (B) Total FOXA2 by western blot, and detection of nuclear FOXA2 by western blot, *n* = 3/group. (C) FOXA2 in fetal lung tissues by immunoprecipitation, p‐FOXA2 was analyzed by western blot, *n* = 3/group. (D) Cell apoptosis using the TUNEL assay. Green fluorescence identifies TUNEL‐positive cells, and blue represents cell nuclei. Bars represent means ± SD; *P <* *0.05 between indicated samples.

Forkhead box A2 was expressed in AECII, as detected by immunofluorescence (Fig. 3A). Western blot showed significantly lower nuclear FOXA2 expression in the DM fetal lungs compared with controls (*P *<* *0.05) (Fig. 3B). Notably, the expression of p‐FOXA2 was significantly higher in the DM fetal lung tissues (*P *<* *0.05) (Fig. 3C). These findings suggested that higher p‐FOXA2 and lower nuclear FOXA2 in DM offspring might be associated with lung dysplasia in rats. While no significant difference in total FOXA2 gene or protein expression was found (Fig. 3B), the activated form of FOXA2 was lower in the DM fetal lungs. Furthermore, the TUNEL assay suggested that the apoptotic ratio was higher in DM offspring than in controls (Fig. 3D).

### MK2206 partly reversed lower nuclear FOXA2 and higher p‐FOXA2 expression observed under high glucose/insulin conditions

**Figure 4:**
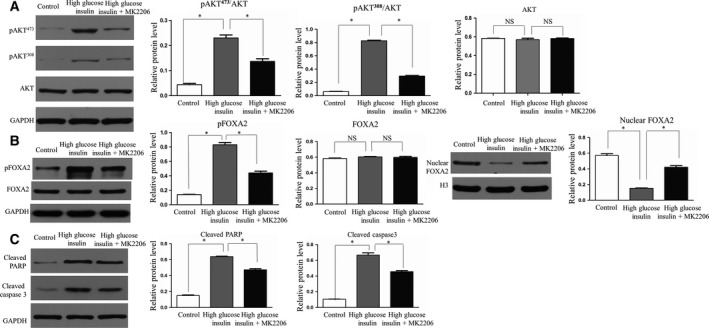
Expression of FOXA2, nuclear FOXA2, and p‐FOXA2 in MLE12 cells with no treatment, high glucose/insulin conditions, and high glucose/insulin conditions combined with MK2206. (A) Phosphorylation (both at p308 and at p473) and total levels of Akt protein were measured by western blot. (B) Protein levels of p‐FOXA2, total FOXA2, and nuclear FOXA2 were measured by western blot. (C) PARP and caspase 3 cleavage were used to determine apoptosis levels. The experiments were performed in triplicate. Bars represent means ± SD; *P <* *0.05 between indicated samples. The experiments were performed in triplicate.

Further testing using an *in vitro* model showed that the Akt pathway was inhibited when an Akt inhibitor was used. The levels of p‐AKT were the highest in the high glucose/insulin group and were decreased when cells under high glucose and insulin condition were treated with MK2206 (*P *<* *0.05) (Fig. 4A). Total FOXA2 protein levels remained at the same level among the groups (*P *>* *0.05) (Fig. 4B). Nuclear FOXA2 extracted from the cells of the high glucose and insulin group was lower than in the control group (*P *<* *0.05). Interestingly, the expression of nuclear FOXA2 was increased in the MK2206 group compared with the high glucose and insulin group (*P *<* *0.05) (Fig. 4B).

The expression of p‐FOXA2 was the highest in the high glucose and insulin group and was decreased when the cells were treated with MK2206 (*P *<* *0.05) (Fig. 4B). The results suggested that changes in nuclear FOXA2 and p‐FOXA2 in cells of the high glucose and insulin group could be partly reversed by MK2206. The analysis of PARP and caspase 3 cleavage suggested that apoptosis was increased compared with controls under high glucose/insulin (*P *<* *0.05), but was decreased by the addition of MK2206 (*P *<* *0.05) (Fig. 4C).

### MK2206 partly reversed the changes in SP‐B and SP‐C expression observed under high glucose and insulin conditions

**Figure 5:**
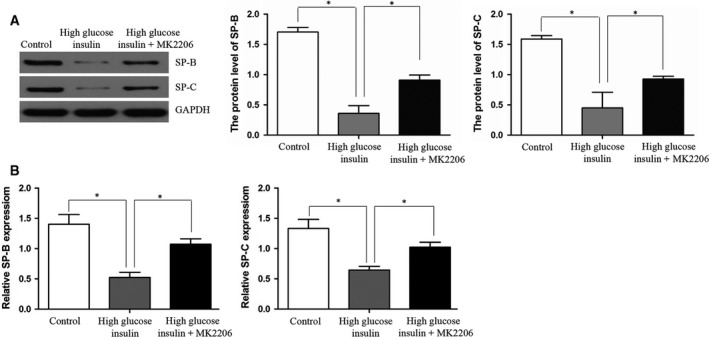
Protein and mRNA expression of SP‐B and SP‐C in MLE12 cells. (A) Protein levels of SP‐B and SP‐C were measured by western blot. (B) Detection of SP‐B and SP‐C mRNA expression by RT‐PCR. Control group: MLE12 without stimulation. High glucose/insulin group: MLE12 treated with glucose and insulin. MK2206 group: MLE12 treated with glucose, insulin, and MK2206. Bars represent means ± SD; *P <* *0.05 between indicated samples. The experiments were performed in triplicate.

Western blot and RT‐PCR showed that the protein and mRNA expression levels of SP‐B and SP‐C in the high glucose and insulin group were lower compared with the control group (*P *<* *0.05), but were increased after inhibition of the Akt pathway by MK‐2206 (*P *<* *0.05) (Fig. 5A,B). The results suggested that lower expression levels of SP‐B and SP‐C in the high glucose and insulin group could be partly reversed by MK2206.

## Discussion

Diabetes exposure is known to impair lung development and to be associated with RDS, but the exact mechanisms are currently unclear. The present study showed that maternal diabetes induces fetal lung FOXA2 phosphorylation through the Akt pathway and also affects the maturation of alveolar epithelial cells, reducing the levels of SP‐B and SP‐C in the fetal lungs. An Akt inhibitor reversed the changes in SP expression *in vitro*.

The lung is affected during fetal development in DM mothers, both in the perinatal period and later in life 34, 35, 36. In the present study, DM fetal lungs showed low markers of fetal lung maturation, as supported by a previous study 37. In addition, the present study suggests that the protein and mRNA expressions of SP‐B and SP‐C in the DM fetal lungs were lower than in controls. The results also showed immature cells with glycogen and lipid deposition, few villi, and few lamellar bodies in the DM fetal lungs. High blood glucose levels stimulate insulin secretion, resulting in fetal hyperinsulinemia 38.

The present study confirmed the expression of FOXA2 in fetal lung epithelial cells. Some studies showed that FOXA2 activates lipid metabolism and ketogenesis in the liver during fasting and is inhibited by insulin‐PI3K‐Akt signaling mediated by phosphorylation at Thr156 and nuclear exclusion, not only during feeding, but also in hyperinsulinemic ob/ob or db/db mice and in animals with diet‐induced obesity 15, 29. The present study showed that nuclear FOXA2 was significantly lower while p‐FOXA2 was higher in the DM fetal lungs compared with controls. Even though there was no change in total FOXA2 expression in fetal lungs, our results suggest that diabetes exposure reduces the activated nuclear FOXA2 through Akt phosphorylation. Supporting these results, a previous study in hyperinsulinemic mice showed that FOXA2 is phosphorylated by Akt via the PI3K‐Akt/PKB pathway, resulting in FOXA2 being released from the nucleus and persistently localized in the cytoplasm, losing transcriptional activity and consequently affecting downstream gene expression 17, 29. Therefore, it could be hypothesized that fetal hyperinsulinemia leads to FOXA2 phosphorylation, and then, p‐FOXA2 is discharged from the nucleus. p‐FOXA2 without transcriptional regulation could then be found in the plasma, affecting the differentiation of AECII and SP synthesis. It is important to note here that the rat diabetes model used in this study induced diabetes early in the pregnancy, and FOXA2 is expressed in AECII late in gestation so there may be some differences between this model and actual gestational diabetes mellitus. There are other differences between short‐ and long‐term activation. Akt has many activities besides regulating glucose metabolism and can become resistant with long‐term diabetes exposure and insulin resistance that normally occurs via Akt may shift to the MAPK pathway 25. In the *in vitro* experiments, in which short‐term high glucose and insulin induction were used, the protein and mRNA expression levels of both SP‐B and SP‐C in the high glucose and insulin group were lower compared with the control group, but were increased by MK2206. The present study confirms that MK2206 reduces the phosphorylation of Akt, as supported by a previous study 27.

This study involved investigation of maternal hyperglycemia, fetal hyperglycemia, and hyperinsulinemia, but has some limitations, the mouse model used STZ to induce β‐cell death and induce diabetes‐like symptoms, but gestational diabetes, the most common type of diabetes during pregnancy, is not due to loss of β‐cell activity 39, so this model may not completely mimic the clinical situation. Nevertheless, our model simulates the important triad that affects fetal development. The maternal insulin levels were measured after anesthesia, which may account for the high levels. Fetal blood was also sampled after maternal chloral hydrate anesthesia and sacrifice. We did not record the sex of the offspring in this study, and gender differences might have an influence on the development of diabetes. We also did not calculate the lung weight/body weight ratio to evaluate whether smaller lungs were found in smaller pups, or count the pups from the uterine horn, since the first four pups are exposed to the highest glucose levels 40, 41, or stratify them by weight, larger pups may be more affected by high glucose levels. These issues should be addressed in future studies.

## Conclusion

In summary, maternal diabetes induces fetal lung FOXA2 phosphorylation through the Akt pathway, thereby affecting the maturation of alveolar epithelial cells and reducing the levels of SP‐B and SP‐C in the fetal lungs. An Akt inhibitor reversed the changes in SP expression *in vitro*. It remains nevertheless to be proven directly that these post‐translational modifications of FOXA2 inactivate its function and cause pulmonary pathologic and cellular evidence of immaturity.

## Author contributions

FTD and QMZ designed the research. FTD, QMZ, and XQC wrote the main manuscript text and prepared Figs 1, 2 and 3. XQC and WXOY prepared Table 1. QMZ and TS prepared Table 2. All authors reviewed the manuscript.
